# Efficacy of Evocalcet in Previously Cinacalcet-Treated Secondary Hyperparathyroidism Patients

**DOI:** 10.1016/j.ekir.2021.08.020

**Published:** 2021-08-23

**Authors:** Fumihiko Koiwa, Shin Tokunaga, Shinji Asada, Yuichi Endo, Masafumi Fukagawa, Tadao Akizawa

**Affiliations:** 1 Division of Nephrology, Department of Internal Medicine, Showa University Fujigaoka Hospital, Yokohama, Japan; 2 Medical Affairs Department, Kyowa Kirin Co., Ltd., Tokyo, Japan; 3 R&D Division, Kyowa Kirin Co., Ltd., Tokyo, Japan; 4 Division of Nephrology, Endocrinology, and Metabolism, Department of Internal Medicine, Tokai University School of Medicine, Kanagawa, Japan; 5 Division of Nephrology, Department of Medicine, Showa University School of Medicine, Tokyo, Japan

**Keywords:** calcium, cinacalcet, evocalcet, parathyroid gland volume, parathyroid hormone, secondary hyperparathyroidism

## Abstract

**Introduction:**

Evocalcet is a recently approved calcimimetic agent for secondary hyperparathyroidism (SHPT). In this study, the efficacy and safety of once-daily oral evocalcet were evaluated in patients without prior cinacalcet use (nonusers) and previously treated patients (users).

**Methods:**

This *post hoc* analysis of a previous phase III head-to-head comparison study included SHPT patients treated with evocalcet with or without prior cinacalcet use. Endpoints included trends in the median intact and whole parathyroid hormone (PTH), mean corrected calcium, phosphate, and bone metabolic markers, and whole-to-intact PTH ratios throughout the 30-week study period; proportions of patients achieving target intact PTH, corrected calcium, and phosphate at weeks 28 to 30; and adverse drug reactions (ADRs).

**Results:**

This study included 127 nonusers and 190 users with significant differences in age; duration of dialysis; use of intravenous vitamin D receptor activators; levels of intact PTH, corrected calcium, tartrate-resistant acid phosphatase 5b, procollagen type 1 N-terminal-propeptide; and largest parathyroid gland volume (*P* < 0.05 for all characteristics) between 2 groups at baseline. Users required higher evocalcet dosages than nonusers. Similar efficacy results were found in the 2 groups except for a significantly higher proportion of nonusers achieving the intact PTH target (81.6% vs 67.1%, difference [95% confidence interval], −14.5% [−24.59, −3.34]), and a significant reduction in largest parathyroid gland volume from week 0 to week 30 (−120.6 [567.2] mm^3^, *P* = 0.043). No difference was found in ADRs between the 2 groups.

**Conclusion:**

Treatment with evocalcet is effective and safe irrespective of prior cinacalcet treatment in SHPT patients.

## Graphical abstract

**Figure:**
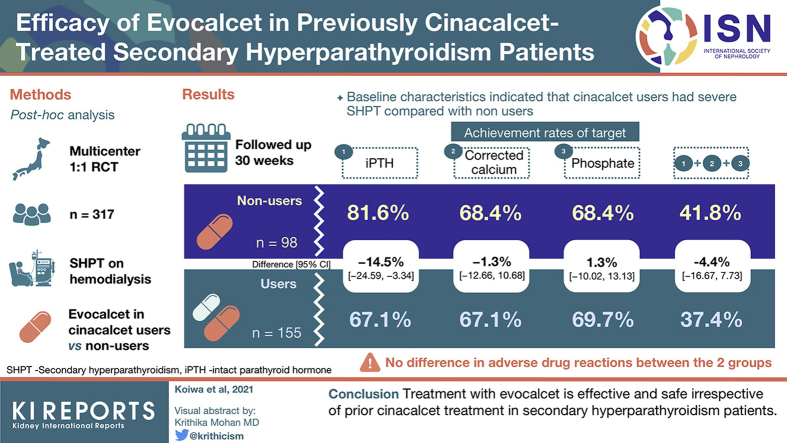

Chronic kidney disease patients requiring hemodialysis often develop secondary hyperparathyroidism (SHPT), which is characterized in part by elevated levels of serum parathyroid hormone (PTH), calcium, and phosphate.^1^, ^2^, ^3^ In addition to hormones and minerals, bone metabolism is affected as indicated by elevated serum bone-specific alkaline phosphatase (BAP), tartrate-resistant acid phosphatase 5b (TRACP-5b), and procollagen type 1 N-terminal propeptide (P1NP) levels, as well as fibroblast growth factor 23 (FGF23) levels, which cause fibro-osteitis and increase the risk of bone/joint pain and bone fractures.^4^, ^5^, ^6^ Calcium and phosphate, released from bones, induce ectopic calcification of the vascular intima and media, which eventually increases the incidence and mortality of cardiovascular diseases.^7^ Therefore, it is critical to control serum levels of PTH, minerals, and bone metabolic markers in SHPT patients undergoing hemodialysis.

In the 2012 revision of the “Clinical Practice Guideline for the Management of Chronic Kidney Disease−Mineral and Bone Disorder” by the Japanese Society for Dialysis Therapy (JSDT), the target ranges for serum PTH (60–240 pg/ml), calcium (8.4–10.0 mg/dl), and phosphate (3.5–6.0 mg/dl) levels were determined from the viewpoint of life prognosis, and treatment guidelines to maintain these levels within the target ranges are recommended.^8^ Traditionally, vitamin D derivatives, phosphate binders, and parathyroidectomy have been treatment options for SHPT.

Cinacalcet was the first calcimimetic agent approved for the treatment of SHPT by the US Food and Drug Administration in 2004.^9^ The proportion of patients who achieved the guideline target ranges for serum PTH, calcium, and phosphate levels were increased with cinacalcet, which indicates that PTH as well as calcium and phosphate control were also improved with cinacalcet. In additionally, since cinacalcet was approved, vitamin D receptor activator (VDRA)−based strategies and the number of patients undergoing parathyroidectomy have decreased dramatically.^10^^,^^11^ Cinacalcet also suppresses the progress of vascular calcification and reduces the risk of cardiovascular complications.^12^

However, patients treated with cinacalcet often experience upper gastrointestinal adverse drug reactions (ADRs) that include vomiting and nausea.^9^ A relatively new calcimimetic agent, evocalcet, decreases PTH levels in a manner similar to that of cinacalcet, but with a lower frequency of upper gastrointestinal ADRs.^13^, ^14^, ^15^, ^16^, ^17^, ^18^, ^19^, ^20^ Therefore, evocalcet is likely to be beneficial for patients who cannot take higher cinacalcet dosages and/or those with low adherence to cinacalcet treatment because of ADRs. Because evocalcet was approved for SHPT, some patients start treatment with evocalcet without prior cinacalcet use (cinacalcet nonusers), whereas others switch from cinacalcet to evocalcet (cinacalcet users). To date, no study has assessed the impact of pretreatment with cinacalcet on the efficacy and safety of evocalcet.

Evocalcet was approved in 2018 and is marketed in Japan only. Currently, there is less clinical evidence for evocalcet compared with cinacalcet; thus, it is critical to obtain more clinical evidence for evocalcet. In this study, *post hoc* analyses were applied to data already available from a previous phase III head-to-head comparison study,^17^ to evaluate the efficacy and safety of evocalcet in cinacalcet nonusers and users.

## Materials and Methods

This study was approved by the institutional review board at study sites, and all patients provided a signed consent form prior to participating in the study. The study was conducted in accordance with the principles of the Declaration of Helsinki, Good Clinical Practice, and applicable local regulations.

### Previous Phase III Head-to-Head Comparison Study

A previous multicenter, active-controlled, randomized, double-blind, double-dummy, intra-patient dose-adjustment, parallel-group study compared the efficacy and safety of once daily oral evocalcet with cinacalcet in patients with SHPT undergoing dialysis, who were randomly allocated 1:1 to receive once daily oral evocalcet or cinacalcet for 30 weeks (phase III head-to-head comparison study) (ClinicalTrials.gov, NCT02549391 and JAPIC, JapicCTI-153013).^17^ The dosage of evocalcet was adjusted in the first 28 weeks (dosage adjustment period, week 0 to week 28), and the efficacies of evocalcet were evaluated at week 28 to week 30 (evaluation period), during which the dosage of evocalcet was fixed. The starting dosage of evocalcet was determined based on the intact PTH level 1 week before the initiation of treatment with evocalcet: patients with intact PTH <500 pg/ml were started at 1 mg/d, and those with ≥500 pg/ml were started at 2 mg/d. The dosage of evocalcet was then adjusted to control the intact PTH level within 60 pg/ml ≤ intact PTH level ≤ 240 pg/ml. The dosage was increased by 1-mg increments up to 8 mg if the same dosage was maintained for ≥3 weeks, the intact PTH level was >240 pg/ml, and the corrected calcium level was ≥8.4 mg/dl. The dosage was decreased if the intact PTH level was <60 pg/ml or if the investigators determined that it was necessary because of ADRs. Treatment with evocalcet was interrupted if the corrected calcium level was ≤7.5 mg/dl or if the investigators determined that it was necessary to interrupt because of ADRs. Treatment with cinacalcet was prohibited for 2 weeks before screening. Intact PTH and whole PTH were measured using an electrochemiluminescence immunoassay (ECLusys PTH, Roche Diagnostics K. K., Tokyo, Japan) and immunoradiometric assay (Whole PTH “Sumitomo”, DS Pharma Biomedical Co., Ltd, Osaka, Japan), respectively.

### Study Design

This study was a *post hoc* analysis of a previous phase III head-to-head comparison study. Patients treated with evocalcet were divided into 2 groups based on prior cinacalcet use (nonusers and users) and subjected to the analyses.

### Endpoints

#### Efficacy Endpoints

The following efficacy endpoints were evaluated in cinacalcet nonusers and users: weekly trends in median intact and whole PTH levels; mean corrected calcium and phosphate levels; trends in median intact FGF23 levels at 3-week intervals; trends in mean BAP, TRACP-5b, and P1NP levels at 6-week intervals; weekly trends in whole-to-intact PTH ratios throughout the 30 weeks of the study period; proportions of nonusers/users who achieved the target ranges for intact PTH, corrected calcium, phosphate, and all 3 at week 28 to week 30 according to the target ranges set by the JSDT,^8^ as well as weekly trends in the proportions of nonusers/users with intact PTH, corrected calcium, and phosphate levels above, within, and below the target ranges throughout the 30 weeks of the study period; mean and percentage changes in largest parathyroid gland volume at week 30 from week 0; and weekly trends in the mean evocalcet dosages throughout the study period.

#### Safety Endpoint

Predetermined hypocalcemia-related ADRs (corrected calcium decreased, blood calcium decreased, and hypocalcemia) and upper gastrointestinal tract–related ADRs (nausea, vomiting, abdominal discomfort, abdominal distension, and decreased appetite) were evaluated in cinacalcet nonusers and users.

### Statistical Analyses

All statistical analyses were conducted using SAS version 9.4 (SAS Institute, Inc., Cary, NC). Statistical significance was set at 2-tailed *P* < 0.05. In this study, the efficacy endpoints were analyzed in the per-protocol set, which included patients who were randomized and treated with at least 1 dose of evocalcet, except those who violated eligibility criteria, were prescribed evocalcet for ≥28 weeks with a medication adherence <70%, were treated with a prohibited concomitant drug and/or therapy, had 2 or more missing intact PTH measurements from 3 time points during the evaluation period (week 28 to week 30), or protocol violation that might have affected the outcome of the efficacy results. The safety endpoint was evaluated in the safety analysis set, which included all randomized patients except those who did not receive even a single dose of evocalcet.

Patient baseline data used in this study were sex, age, height, body weight, body mass index, primary disease (diabetic nephropathy, chronic glomerulonephritis, nephrosclerosis, and other), duration of dialysis, type of dialysis (hemodialysis or hemodiafiltration), dry weight, dialysis efficiency, concomitant medications at week 0, VDRA use at week 0 (oral only or oral and i.v.), serum hormone and mineral levels (intact and whole PTH, corrected calcium, phosphate, corrected calcium−phosphate product, BAP, TRACP-5b, P1NP, and intact FGF23), and largest parathyroid gland volume. Categorical data were presented as frequency or percentage, and continuous data were presented as the number of patients and percentage. Intact and whole PTH, and intact FGF23 levels were presented as the median (interquartile range [IQR]), and others as the mean (SD). Statistical differences for categorical variables were estimated using the χ^2^ test, and continuous variables were assessed using the unpaired *t* test and Mann–Whitney *U* test.

For efficacy endpoints, the proportion and 95% confidence interval (CI) of patients achieving the target ranges of intact PTH, corrected calcium, and phosphate were calculated. Analyses of largest parathyroid gland volume included patients who had measurement data at week 0 and week 30. The mean changes and percentage changes in largest parathyroid gland volume were compared using the Wilcoxon signed-rank test. For trends in mean evocalcet dosages, the first dosage was used if it was changed within 1 week.

Adverse drug reactions were monitored over the entire study period and classified using the Medical Dictionary for Regulatory Activities version 19.0. Incidences of pre-determined ADRs were evaluated in cinacalcet nonusers and users.

## Results

### Patient Demographic and Clinical Baseline Data

Overall, 320 patients were randomized to receive evocalcet in the previous head-to-head comparison study, of whom 3 patients did not receive evocalcet by a randomized method; therefore, 317 patients were included in the safety analysis set (127 cinacalcet nonusers and 190 users; hereinafter in this order),^17^ and 253 patients (98 nonusers and 155 users) were included in the per-protocol set.

At baseline, the mean (SD) age was 60.9 (11.4) years, and 70.0% of the patients were male. Common primary diseases included chronic glomerulonephritis (41.1%) and diabetic nephropathy (26.1%). Most patients were on hemodialysis (79.1%) and treated with i.v. VDRA (53.8%). When patient baseline characteristics were compared between cinacalcet nonusers and users, significant differences were found for age, duration of dialysis, use of i.v. VDRA, intact PTH, corrected calcium, TRACP-5b and P1NP levels, and largest parathyroid gland volume (*P* < 0.05 for all characteristics, χ^2^ test [categorical], unpaired *t* test or Mann–Whitney *U* test [continuous]) (Table 1), which indicated that cinacalcet users had severe SHPT compared with nonusers.

**Table 1:** Patient baseline characteristics

Parameter	All patients	Prior cinacalcet use		*P* value
		Nonusers	Users	
	N = 253	n = 98	n = 155	
Sex				
Male	177 (70.0)	69 (70.4)	108 (69.7)	0.902
Age, yr	60.9 ± 11.4	63.8 ± 11.3	59.2 ± 11.1	0.002
Body mass index, kg/m^2^	24.6 ± 4.6	25.0 ± 4.5	24.3 ± 4.6	0.202
Primary disease				
Diabetic nephropathy	66 (26.1)	27 (27.6)	39 (25.2)	0.560
Chronic glomerulonephritis	104 (41.1)	35 (35.7)	69 (44.5)	
Nephrosclerosis	31 (12.3)	13 (13.3)	18 (11.6)	
Other	52 (20.6)	23 (23.5)	29 (18.7)	
Duration of dialysis, mo	126.5 ± 86.2	92.6 ± 70.9	147.9 ± 88.3	< 0.001
Type of dialysis				
HD	200 (79.1)	81 (82.7)	119 (76.8)	0.263
HDF	53 (20.9)	17 (17.3)	36 (23.2)	
Dry weight, kg	62.2 ± 13.9	63.3 ± 14.2	61.6 ± 13.7	0.342
Dialysis efficiency, spKt/V	1.50 ± 0.29	1.46 ± 0.28	1.52 ± 0.29	0.092
Use of vitamin D receptor activator				
Users	217 (85.8)	80 (81.6)	137 (88.4)	0.134
Oral only	81 (32.0)	38 (38.8)	43 (27.7)	0.018
Parenteral	136 (53.8)	42 (42.9)	94 (60.6)	
Intact PTH, pg/ml	374 (298, 472)	336 (278, 415)	390 (311, 511)	0.002
Corrected calcium, mg/dl	9.5 ± 0.6	9.3 ± 0.5	9.6 ± 0.6	< 0.001
Phosphate, mg/dl	5.8 ± 1.3	5.7 ± 1.4	5.8 ± 1.3	0.448
Corrected calcium−phosphate product, mg^2^/dl^2^	54.6 ± 12.3	52.9 ± 12.6	55.7 ± 12.1	0.073
Bone-specific alkaline phosphatase, μg/l	17.6 ± 9.9	17.0 ± 8.6	17.9 ± 10.7	0.744
Tartrate-resistant acid phosphatase 5b, mU/dl	783.2 ± 394.5	682.3 ± 348.7	846.9 ± 409.3	< 0.001
Procollagen type 1 N-terminal-propeptide, μg/l	437.3 ± 287.5	363.4 ± 233.1	484.0 ± 308.8	0.002
Fibroblast growth factor 23, pg/ml	13 600 (6130, 28 600)	12 150 (5010, 24 200)	15 300 (6640, 30 700)	0.120
Largest parathyroid gland volume, mm^3^	332.1 ± 627.1	290.6 ± 613.1	354.7 ± 636.4	0.025

Data are number (%), mean ± SD, or median (interquartile range). ꭓ^2^ Test (categorical), unpaired *t* test or Mann–Whitney *U* test (continuous). HD, hemodialysis; HDF, hemodiafiltration; PTH, parathyroid hormone.

Patients in both groups started to receive similar dosages of evocalcet at week 1 (Supplementary Figure S1); however, over the dosage adjustment period, the evocalcet dosage in the users (>4 mg/d) increased more than that in the nonusers (approximately 2.6 mg/d).

### Efficacy

**Figure 1:**
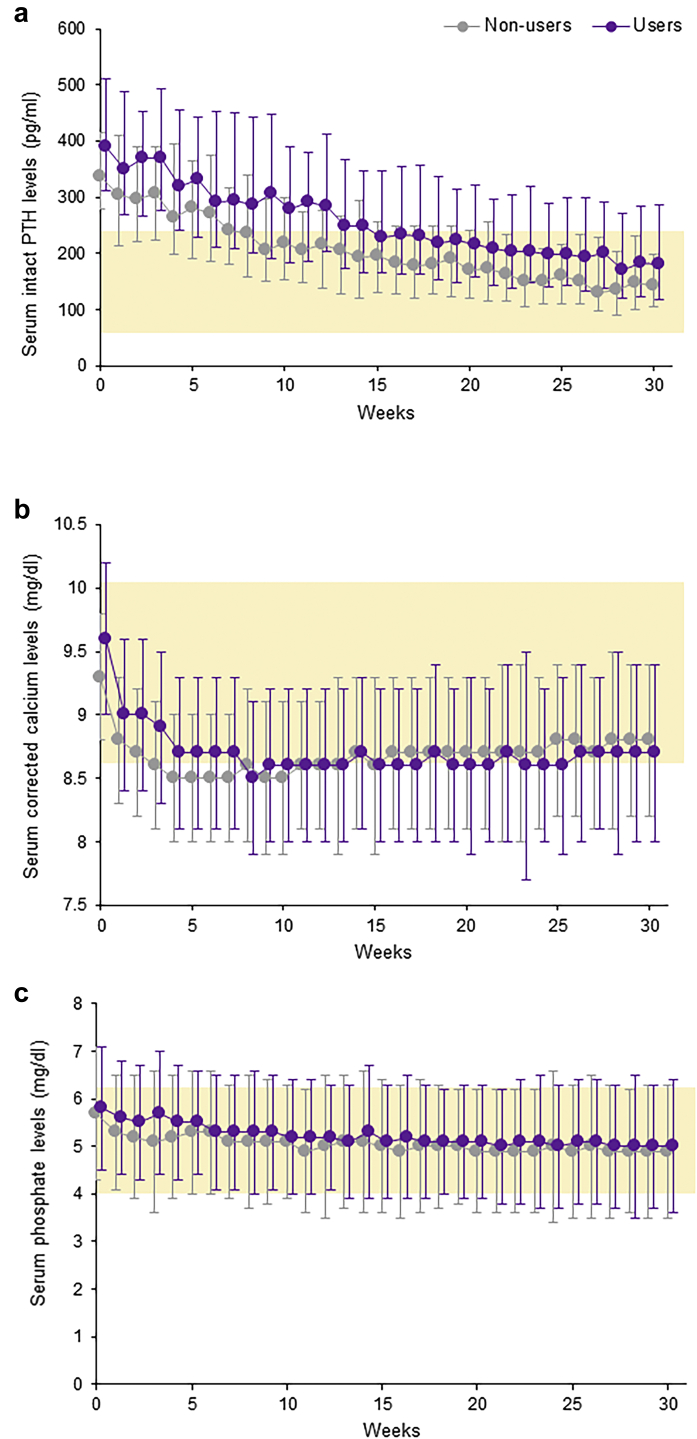
Trends in the (a) median (interquartile range) serum intact PTH levels, (b) mean (SD) serum corrected calcium, and (c) serum phosphate levels throughout the study period (week 0 to week 30) in cinacalcet nonusers and users. Yellow-shaded area indicates a guideline target range. PTH, parathyroid hormone.

Similar declining trends were observed in both groups for intact PTH, corrected calcium, and phosphate levels over the 30 weeks of treatment with evocalcet, irrespective of prior cinacalcet treatment (Figure 1). At week 0, the median (IQR) intact PTH levels in the nonusers (336 [278, 415] pg/ml) and the users (390 [311, 511] pg/ml) were above the guideline target range, but they both decreased to within the target range after 30 weeks of treatment with evocalcet (142 pg/ml and 182 pg/ml, respectively) (Figure 1a). The median whole PTH level in both groups showed trends similar to those of the intact PTH level (Supplementary Figure S2A). Indeed, whole-to-intact PTH ratios were constant in both groups throughout the study period (Supplementary Figure S2B).

The mean corrected calcium level in both groups was decreased in the first 4 weeks and then maintained throughout the rest of the study period (Figure 1b). The mean phosphate level in both groups gradually decreased over the 30 weeks of the study period (Figure 1c).

**Figure 2:**
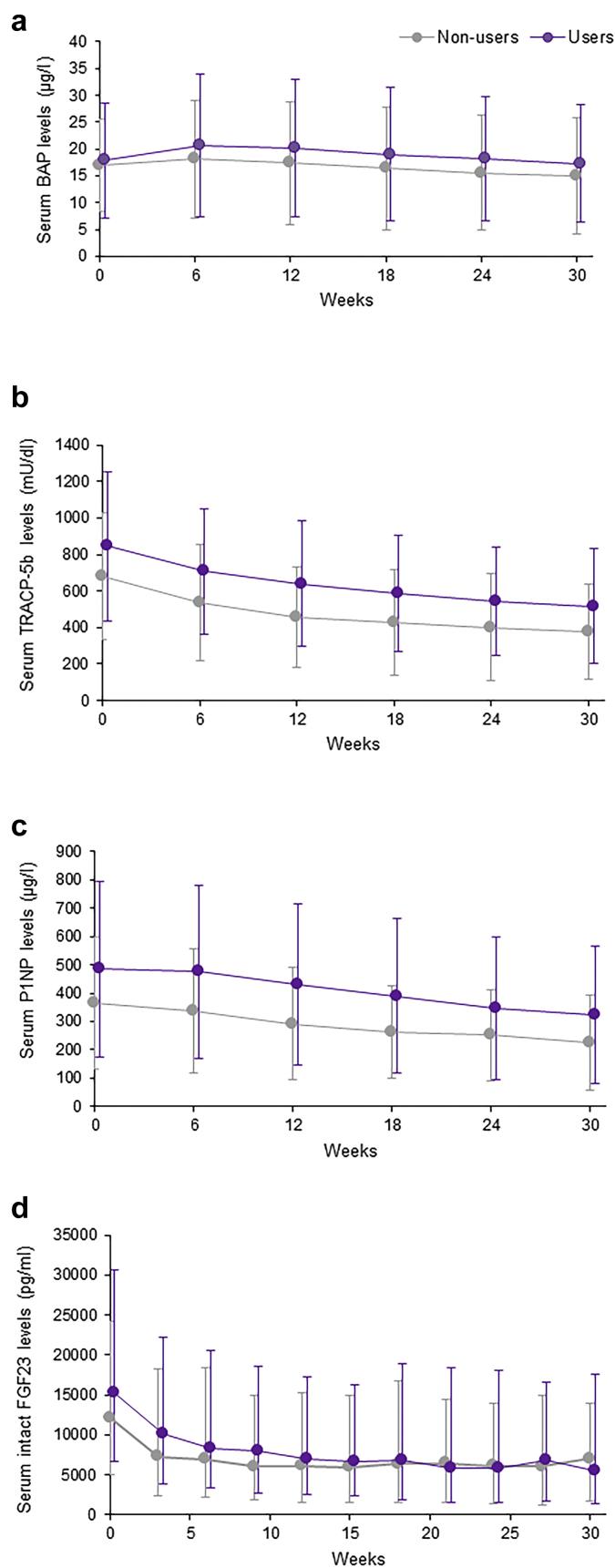
Trends in the mean (standard deviation) serum (a) BAP, (b) TRACP-5b, and (c) P1NP, and median (interquartile range) intact (d) FGF23 levels throughout the study period (week 0 to week 30) in cinacalcet nonusers and users. BAP, bone-specific alkaline phosphatase; FGF23, fibroblast growth factor 23; P1NP, procollagen type 1 N-terminal propeptide; TRACP-5b, tartrate-resistant acid phosphatase 5b.

As shown in Figure 2, the mean levels of bone metabolic markers, BAP, TRACP-5b, and P1NP, decreased over the 30 weeks of treatment with evocalcet, irrespective of prior cinacalcet treatment. In addition, the median intact FGF23 level showed a similar decline in both groups (Figure 2d).

The proportions of nonusers/users who achieved the guideline target range for intact PTH, corrected calcium, phosphate, and all 3 during the evaluation period (week 28 to week 30) in the 2 groups are summarized in Table 2. A significantly higher proportion of nonusers (81.6%) achieved the intact PTH target range compared with users (67.1%) (difference [95% CI], −14.5% [−24.59, −3.34]). However, no significant difference was found for the corrected calcium target (68.4% vs. 67.1%) or phosphate (68.4% vs. 69.7%) between the 2 groups. The proportion of nonusers who achieved all 3 targets (41.8%) was similar to that of the users (37.4%) during the evaluation period. Over the 30 weeks of treatment, the proportion of nonusers who achieved the target intact PTH level was higher than that of the users, and those with intact PTH levels above the target range were lower than that of the users (Supplementary Figure S3). No obvious difference in the proportions of patients whose corrected calcium and phosphate levels were above, within, or below the respective target ranges were observed between the 2 groups over the 30 weeks of treatment.

**Table 2:** Achievement rates of target intact PTH, corrected calcium, and phosphorus levels during the evaluation period (week 28 to week 30) by prior cinacalcet use

	Prior cinacalcet use	
	Nonusers	Users
	n = 98	n = 155
Intact PTH		
n (%)	80 (81.6)	104 (67.1)
Difference (95% CI)		–14.5 (–24.59, –3.34)
Corrected calcium		
n (%)	67 (68.4)	104 (67.1)
Difference (95% CI)		–1.3 (–12.66, 10.68)
Phosphate		
n (%)	67 (68.4)	108 (69.7)
Difference (95% CI)		1.3 (–10.02, 13.13)
Intact PTH, corrected calcium and phosphate		
n (%)	41 (41.8)	58 (37.4)
Difference (95% CI)		–4.4 (–16.67, 7.73)

CI, confidence interval; PTH, parathyroid hormone.

Measurements of largest parathyroid gland volume at week 0 and week 30 were obtained in 56 nonusers and 103 users (Table 3). The mean (SD) largest parathyroid gland volume in nonusers was significantly decreased from week 0 to week 30 (−120.6 [567.2] mm^3^, *P* = 0.043, Wilcoxon signed-rank test), but the mean (SD) percentage change (−1.7% [54.4%]) did not reach statistical significance. There was no significant mean change or percentage change in largest parathyroid gland volume in users.

**Table 3:** Change and percentage change in the largest volume of parathyroid gland from baseline at week 30

Largest volume of parathyroid gland	Prior cinacalcet use	n	Mean ± SD	*P* value
Change (mm^3^)	All	159	–39.3 ± 355.8	0.091
	Nonusers	56	–120.6 ± 567.2	0.043
	Users	103	4.9 ± 130.5	0.680
Percentage change (%)	All	159	–0.3 ± 50.8	0.196
	Nonusers	56	–1.7 ± 54.4	0.510
	Users	103	0.5 ± 48.9	0.275

Wilcoxon signed-rank test.

### Safety

There was no significant difference in the incidence of hypocalcemia-related ADRs between the 2 groups (Supplementary Table S1). Although the incidence of decreased corrected calcium was slightly higher in nonusers compared with users, this was not significantly different. The incidence of upper gastrointestinal tract–related ADRs was not significantly different between the 2 groups.

## Discussion

Currently, no study has assessed the impact of pretreatment with cinacalcet on the efficacy and safety of evocalcet. We therefore designed and conducted this *post hoc* analysis using a previous phase III head-to-head comparison study to evaluate the efficacy and safety of evocalcet in cinacalcet nonusers and users. The results of this study demonstrated that treatment with evocalcet is efficacious and safe in SHPT patients, irrespective of prior cinacalcet treatment. At baseline, a higher proportion of users were treated with i.v. VDRA and had high levels of intact PTH, corrected calcium, and bone metabolic markers, as well as high largest parathyroid gland volume compared with nonusers. Furthermore, users were treated with higher dosages of evocalcet than nonusers. Evocalcet mediates its effects via extracellular calcium-sensing receptors by increasing their sensitivity to calcium, which eventually suppresses serum PTH levels. As previously reported, the sensitivity of calcium-sensing receptors to calcium in patients with more severe SHPT (e.g., those with an enlarged parathyroid gland) was decreased.^21^ Therefore, patients with severe SHPT are likely to require a high dosage of a calcimimetic agent to control serum PTH and mineral marker levels. Although the effects of a 2-week cinacalcet wash-out period are not completely negligible, the present and previous results indicate that users in the present analyses had more severe SHPT at baseline compared with nonusers.

In the previous phase III head-to-head comparison study, the proportion of patients who achieved the target levels of intact PTH, corrected calcium, phosphate, and all 3 but not weekly trends in the intact PTH, whole PTH, corrected calcium, serum-ionized calcium, phosphorus, and intact FGF23 levels, was statistically analyzed between the evocalcet and cinacalcet groups because of differences in the baseline characteristics and in the dose adjustment patterns for cinacalcet and evocalcet.

The present results showed that after 30 weeks of treatment with evocalcet, the median intact PTH levels fell within the guideline target range in both groups, and that the corrected calcium and phosphate levels were similarly controlled in the 2 groups. The levels of bone metabolic markers and FGF23 were also decreased in both groups, irrespective of prior cinacalcet use. These results indicate that, despite the difference in the severity of SHPT, treatment with evocalcet improved the serum levels of PTH, mineral, and bone metabolic markers in both groups.

However, a significantly higher proportion of nonusers achieved the intact PTH guideline target range compared with users. Conversely, the 30-week trend for proportions of patients above, within, and below the target range for calcium and phosphate levels were similar in the 2 groups. Therefore, this suggests that the sensitivity of extracellular calcium-sensing receptors in the parathyroid glands of users might be decreased, possibly because they had a more advanced stage of disease compared with nonusers. Patients included in the present analyses were treated with evocalcet up to 8 mg for 30 weeks in the previous phase III head-to-head comparison study.^17^ However, the approved dosage of evocalcet for the treatment of SHPT is up to 12 mg, which suggests that further reductions in intact PTH and, therefore, a higher guideline target achievement rate, might be expected even in users if they were treated at higher dosages for more than 30 weeks. Indeed, a previous study of 52 weeks of treatment with evocalcet up to 12 mg showed further improvement in serum PTH levels with no increased safety concerns.^22^ More studies are needed to verify the long-term efficacy and safety of evocalcet in real clinical settings in which patients may often switch from cinacalcet to evocalcet.

In this study, a significant reduction in largest parathyroid gland volume from week 0 to week 30 was observed in nonusers. Because the severity of SHPT in patients between the 2 groups was different at baseline, it is difficult to directly compare the efficacy of evocalcet on the largest parathyroid gland volume between the 2 groups. However, our results showed that evocalcet significantly reduced the largest parathyroid gland volume in nonusers, which suggests that evocalcet effectively reduces the largest parathyroid gland volume if patients start the treatment during the early stages of SHPT. As previously demonstrated with cinacalcet, a longer treatment duration might further reduce the volume of parathyroid glands.^23^ Therefore, it would be more practical and efficient to initiate treatment with evocalcet during the early stages of SHPT to decrease the level of intact PTH and the largest parathyroid gland volume.

The intact PTH assay measures whole PTH(1–84) as well as the PTH(7–84) fragment; therefore, it may overestimate or underestimate the level of actual biologically active whole PTH(1–84).^24^ As shown in previous studies, the secretion of PTH(1–84) and PTH(7–84) from parathyroid cells are regulated differently: PTH(1–84) is secreted more under conditions of hypocalcemia, and PTH(7–84) is secreted more under conditions of hypercalcemia.^25^, ^26^, ^27^ Because evocalcet decreases serum calcium levels, it may affect the levels of whole and intact PTH differently. Therefore, in this study, we measured the level of whole PTH and assessed the whole-to-intact PTH ratios. Our results showed that the levels of whole PTH decreased over 30 weeks of treatment in both groups, similar to that for intact PTH. In addition, the baseline whole-to-intact PTH ratios in nonusers and users were consistent with previous studies.^24^^,^^28^^,^^29^ The whole-to-intact PTH ratio was constant over the 30 weeks of treatment with evocalcet, irrespective of prior cinacalcet treatment, which indicates that the ratio was not significantly affected by evocalcet. These results are consistent with a previous study of cinacalcet.^29^ Although more studies are needed to verify the long-term effect of evocalcet, this study suggests that evocalcet decreases whole and intact PTH levels in nonusers and users similarly, without affecting the whole-to-intact PTH ratios.

Some study limitations should be considered when interpreting the present results. First, the patient baseline characteristics showed significant differences between the 2 groups that indicated that users had more severe SHPT at baseline. However, our results demonstrated that evocalcet in users was as effective and safe as in nonusers, which indicates its clinical significance. Second, this study was limited because it was a *post hoc* analysis of a previous clinical study with a limited number of patients; therefore, the results should be interpreted as preliminary findings. In the previous head-to-head comparison study, cinacalcet users were prohibited from taking cinacalcet for 2 weeks prior to enrollment. Therefore, the present analyses were not performed to determine the efficacy and safety of evocalcet when switching from cinacalcet. Two weeks of wash-out might have led to the higher serum levels of baseline intact PTH, corrected calcium, TRACP-5b, and P1NP in those patients. Future studies should evaluate the efficacy, and especially the safety, of evocalcet when it is switched from cinacalcet without a wash-out period. In the previous head-to-head comparison study, patients were prohibited to change or to start treatment with VDRA, whereas they could change treatment to calcium-based/non−calcium-based phosphate binders, which suggests that phosphate binders might have affected the results of the corrected calcium and phosphate levels. Another limitation is that only patients who had data at baseline and week 30 were included in the analysis of largest volume of parathyroid gland, which resulted in a smaller sample size. The last limitation is that only Japanese patients were included, meaning that the present results may not be generalized or applicable to other ethnic groups. Because evocalcet is a relatively new calcimimetic agent, more studies including other ethnic groups should be initiated in the future.

In conclusion, the present results demonstrated that treatment with evocalcet is effective and safe, irrespective of prior cinacalcet treatment. Considering the concern regarding the long-term exposure to the high PTH and mineral levels, our preliminary findings based on this *post hoc* analysis suggest that it may be better if patients start treatment with evocalcet in the early stages of SHPT.

## Disclosure

FK received lecture fees from Kyowa Kirin Co., Ltd. (KKC) and Kissei Pharmaceutical. ST, SA, and YE are employees of KKC. MF received consulting fees from KKC and Ono Pharmaceutical, and lecture fees from KKC, Bayer, Torii Pharmaceutical, and Ono Pharmaceutical; and grants from KKC and Bayer. TA received consulting fees from KKC, Astellas Pharma, Bayer, Fuso Pharmaceutical, Japan Tobacco, Ono Pharmaceutical, Sanwa Chemical, Otsuka, GSK, and NIPRO, and lecture fees from KKC, Chugai Pharmaceutical, Bayer, Kissei Pharmaceutical, Torii Pharmaceutical, and Ono Pharmaceutical.

## Supplementary Material

Supplementary File (PDF)

**Table S1.** Summary of hypocalcemia and upper gastrointestinal tract–related adverse drug reactions

**Figure S1.** Trends in the mean oral evocalcet dosage from week 0 to week 30 in cinacalcet nonusers and users.

**Figure S2.** Trends in median serum whole PTH levels (A) and whole-to-intact PTH ratios (B) throughout the study period (week 0 to week 30) in cinacalcet nonusers and users. PTH, parathyroid hormone.

**Figure S3.** Trends in the proportions of cinacalcet nonusers (left) and users (right) with intact PTH, corrected calcium, and phosphate levels above, within, and below the guideline target ranges throughout the 30 weeks of the study period. PTH, parathyroid hormone.

**List of Collaborators**
