# Supplementary material for: Efficacy of Evocalcet in Previously Cinacalcet-Treated Secondary Hyperparathyroidism Patients
Source: Kidney Int Rep. 2021 Aug 23;6(11):2830–9. doi: 10.1016/j.ekir.2021.08.020 (PMC8589700; doi:10.1016/j.ekir.2021.08.020)
Supplement: Supplementary File (PDF) [file mmc1.pdf]

Table S1. Summary of hypocalcemia and upper gastrointestinal tract-related adverse drug reactions

	Prior cinacalcet use			
	Non-users	Users	Total	
	N = 127	N = 190	N = 317	*P*-value
	n (%)	n (%)	n (%)	
Hypocalcemia-related adverse drug reactions	28 (22.0)	31 (16.3)	59 (18.6)	0.199
Corrected calcium decreased	19 (15.0)	18 (9.5)	37 (11.7)	0.136
Blood calcium decreased	4 (3.1)	7 (3.7)	11 (3.5)	0.799
Hypocalcemia	5 (3.9)	6 (3.2)	11 (3.5)	0.710
Upper gastrointestinal tract-related adverse drug reactions	14 (11.0)	27 (14.2)	41 (12.9)	0.407
Nausea	7 (5.5)	9 (4.7)	16 (5.0)	0.757
Vomiting	3 (2.4)	11 (5.8)	14 (4.4)	0.146
Abdominal discomfort	3 (2.4)	7 (3.7)	10 (3.2)	0.509

Abdominal distension	0 (0.0)	2 (1.1)	2 (0.6)	0.246
Decreased appetite	3 (2.4)	5 (2.6)	8 (2.5)	0.881

χ^2 test.

Figure S1 Trends in the mean oral evocalcet dosage from week 0 to week 30 in cinacalcet non-users and users.

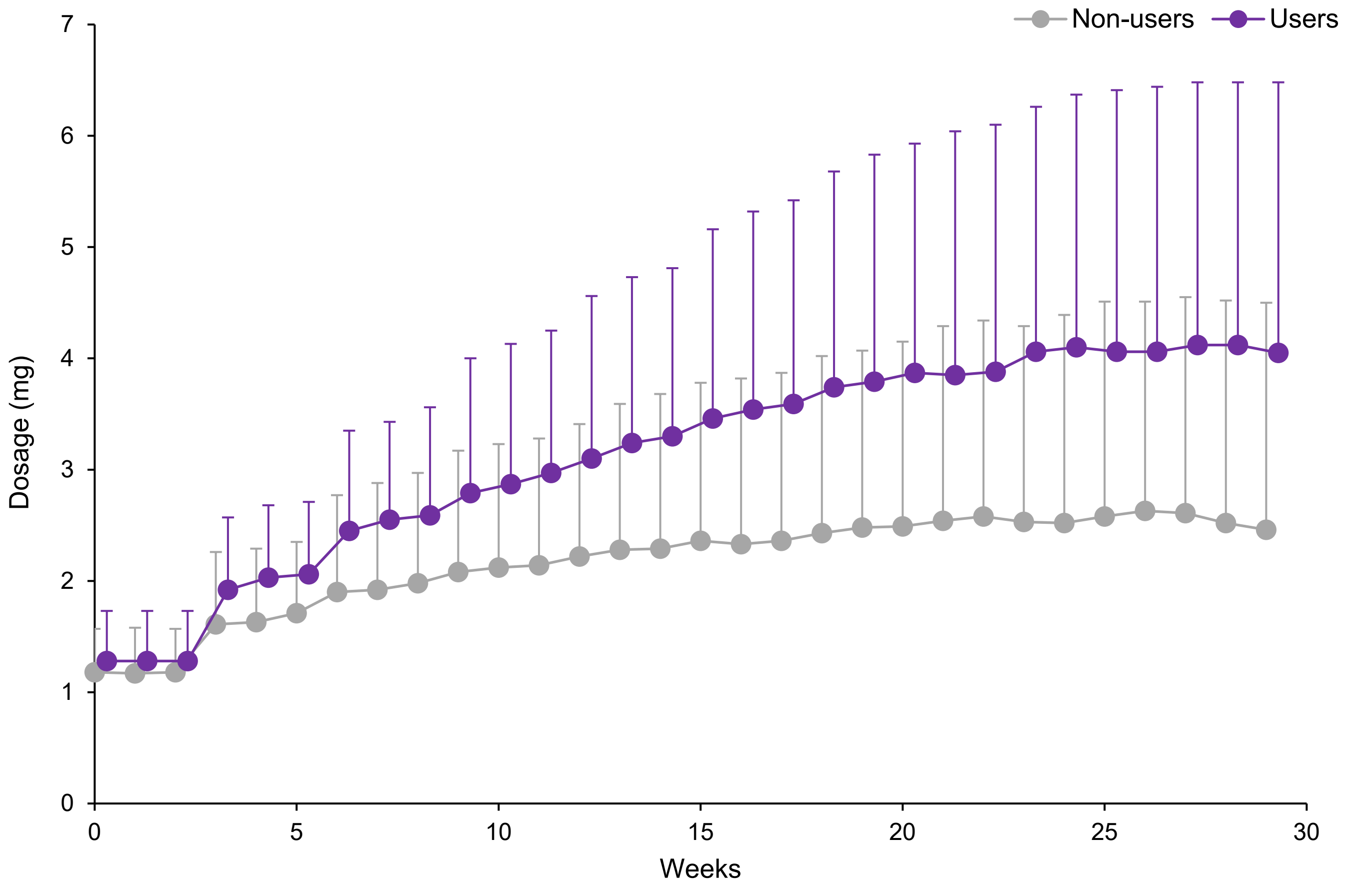

Figure S2 Trends in median serum whole PTH levels (a) and whole-to-intact PTH ratios (b) throughout the study period (week 0 to week 30) in cinacalcet non-users and users. PTH, parathyroid hormone.

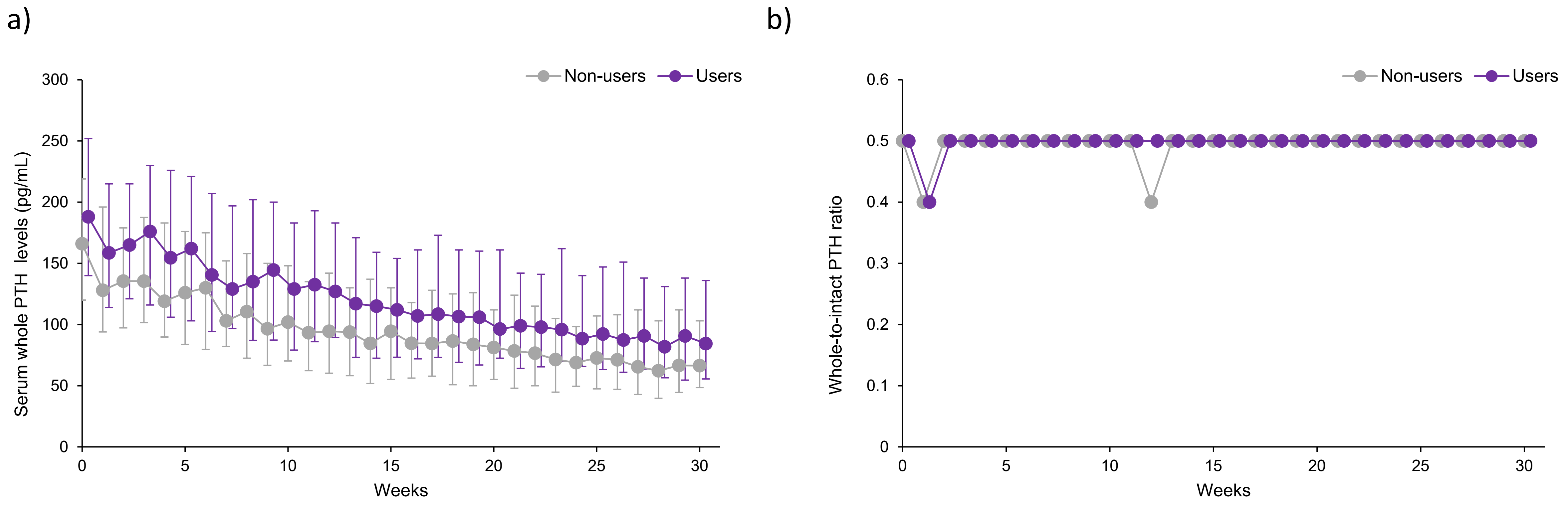

Figure S3 Trends in the proportions of cinacalcet non-users (left) and users (right) with intact PTH, corrected calcium, and phosphate levels above, within, and below the guideline target ranges throughout the 30 weeks of the study period. PTH, parathyroid hormone.

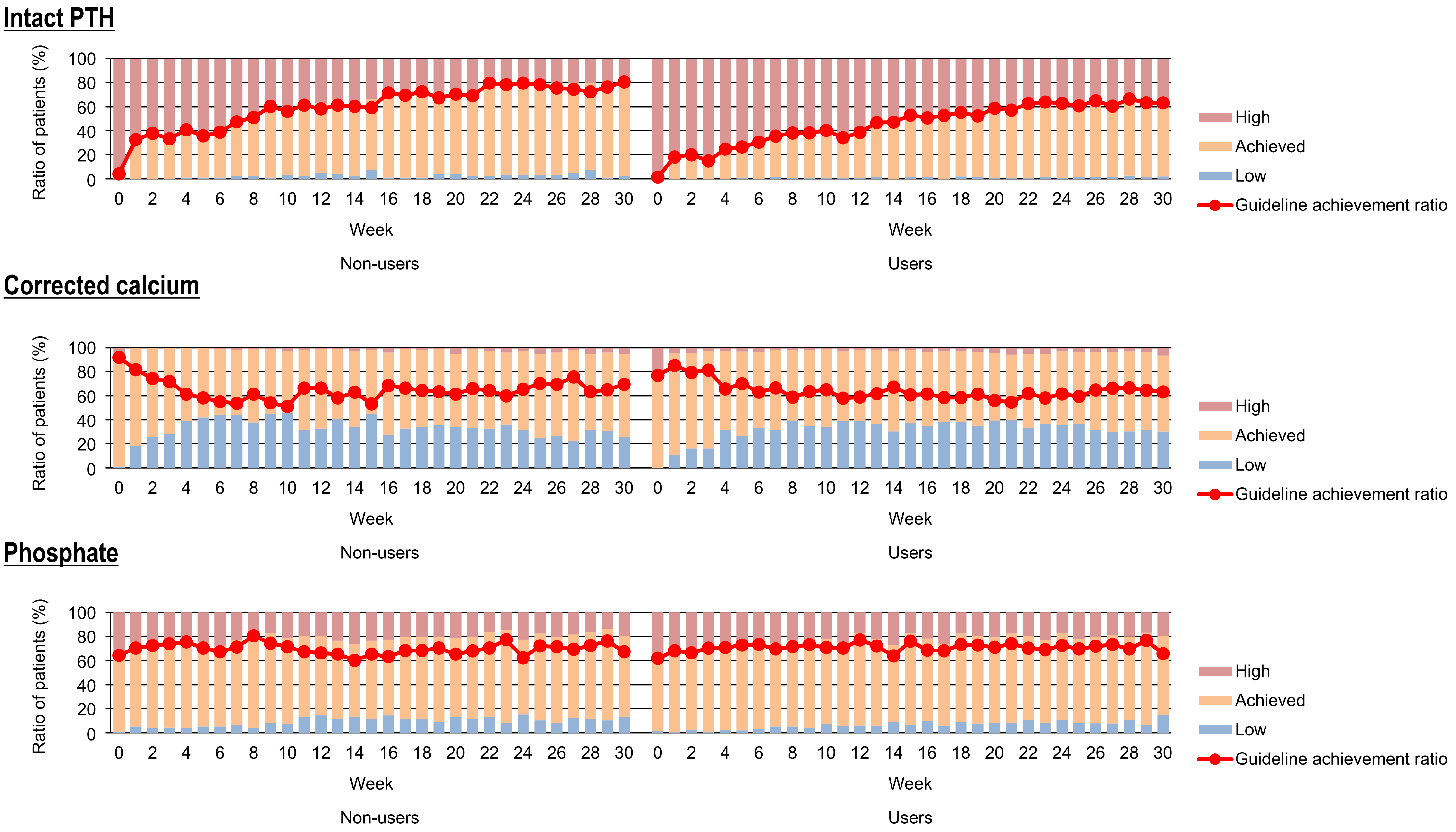

List of Collaborators

Matsugase Yasukuni (Hokkaido P.W.F.A.C. Asahikawa-Kosei General Hospital), Furuta Takashi (Dainohara Clinic), Okazaki Masayuki (Division of Nephrology, Jyoban Hospital, Tokiwakai Group), Otsuka Masakazu (Tokiwa Clinic), Mori Masaru (Tsuchiura Beryl Clinic), Kikuchi Hiroshi (Kikuchi Medical Clinic), Oguchi Kenichi (Renal Unit, Ikegami General Hospital, Medical Corporation Showakai), Oshima Johji (Kubojima Clinic), Enomoto Miho (Ayase Ekimae Jin Clinic), Iwasaki Mitsuko (Department of Nephrology, Toshiba Rinkan Hospital), Suzuki Nobuyuki (Tachibanadai Hospital), Nakakoji Yayoi (Bosei Fujisawa Clinic, Medical Corporation Showakai), Suga Takao (Bosei Hiratsuka Clinic, Medical Corporation Showakai), Kanno Yutaka (Kanno Dialysis & Vascular Access Clinic), Sakai Hiromasa (Fuyo Association Seirei Numazu), Yoshioka Tetsuro (Takeda Hospital Group), Kondo Morihiro (Department of Nephrology, Rakuwakai Otowa Memorial Hospital), Tsujimoto Yoshihiro (Department of Internal Medicine, Inoue Hospital), Nagase Norio (Kawashima Hospital Group), Funakoshi Satoshi (Nagasaki Kidney Center), Oku Manei (Division of Nephrology, Seijinkai Ikeda Hospital), Yoshida Yuichi (Houseikai Higashi Naebo Hospital), Morozumi Kunio (Department of Nephrology, Masuko Memorial Hospital), Yamazaki Chikao (Masuko Hospital Subal), Horie Masanobu (Department of Urology, Daiyukaidaiichi Hospital), Miyata Masahiro (Rifunonaika Clinic), Horigome Ikuo (Kawadaira Medical Clinic), Iitsuka Tadashi (Department of Internal Medicine, Ibaraki Seinan Medical Center Hospital), Oishi Akira (Ohichi Naika Clinic), Ito Kyoko (Heisei Hidaka Clinic), Morioka Junichiro (Ikokai Ora Hospital), Otsuka Keiichi (Oshima Clinic), Miyauchi Yoshihiro (Department of Internal Medicine, Asahi General Hospital), Kaneko Masashi (Kohitsujikai Naganuma Clinic), Takahashi Toshimasa (Bosei Shinjuku Minamiguchi Clinic), Imai Toshikazu (Ken-ei-kai Futakotamagawa Station Clinic), Komatsu Yasuhiro (Nephrology Department, St. Luke's International Hospital), Omata Momoyo (Division of Hemodialysis, Hachioji Azumacho Clinic),

Sugano Yasuji (Kita Hachiouji Clinic), Ogawa Chie (Maeda Institute of Renal Research Musashikosugi Clinic), Kanda Fumiyoshi (Maeda Institute of Renal Research Shin-Yokohama Clinic), Ozawa Kiyoshi (The Department of Internal Medicine, Yokosuka Kyosai Hospital), Nakanishi Taichi (Shinjinkai Kurihama Clinic), Nagaoka Mikako (Boseikai Honatsugimedikaru Clinic), Miyazaki Shigeru (Department of Nephrology, Kidney Center, Shinrakuen Hospital), Tachibana Naoki (Japanese Red Cross Society Suwa Hospital), Oguchi Tomomasa (Department of Internal Medicine, Aizawa Hospital), Okada Yoichi (Maruyamakai Ueda Dialysis Clinic), Hibi Ikuo (Miyaji Clinic), Tsuruta Yoshinari (Meiyo Clinic Hemodialysis Center), Tsuboi Masato (Anjyo Kyoritsu Clinic), Kim Jong Il (Division of Hemodialysis Chibune General Hospital), Nozaki Kanji (Nozaki Clinic), Nishikawa Keiichiro (Fuchu Clinic), Kato Yoshikazu (Shirasagi Hospital), Fukushima Masaki (Department of Internal Medicine, Shigei Medical Research Hospital), Arimoto Katsuhiko (Sowakai Shigei Hospital), Moriishi Misaki (Akane-Foundation Tsuchiya Hospital), Kawai Toru (Chuou Naika Clinic), Obayashi Seiichi (Department of Internal Medicine, Kinashi Obayashi Hospital), Fujii Zenzo (Department of Nephrology, St. Hill Hospital), Mitsui Hiroshi (Department of Urology and Nephrology, Tokuyama Central Hospital), Takayama Kuniko (Takayama Hospital), Tanaka Motoko (Department of Nephrology, Akebono Clinic), Shimoji Kunihiro (Tomishiro Central Hospital), Nakazawa Ryoichi (Department of Drugs, Tokatsu Clinic Hospital, Department of Nephrology), Masuko Hiroyuki (Medical Corporation Bokoi, Nikko Memorial Hospital), Kukita Kazutaka (Sapporo Hokuyu Hospital), Sekino Makoto (Chuou Clinic), Urae Jun (Kojinkai Hospital), Kurihara Satoshi (Saitama Tsukinomori Clinic, Division of Nephrology), Murotani Noriyoshi (Japan Community Health Care Organization, Chiba Hospital), Takaichi Kenmei (Kidney Center, Toranomon Hospital), Shishido Kanji (Kawasaki Clinic), Nakayama Fumiyoshi (Souwa-kai Koshikawa Memorial Yokohama Renal Clinic), Tsukada Wataru (Jishyukai Ueda Kidney Clinic), Nakamura Kazuyoshi (Fuji Daiichi Clinic), Shibata Masaya

(Toyohashi Mates Clinic), Imada Naoki (Department of Urology, Nishijin Hospital), Hashimoto Tetsuya (Touzinkai Hospital), Mikami Satoshi (General Hospital Higashikouri), Usui Koji (Ichiyokai Clinic), Hashimoto Nobuo (H.N. MEDIC), Ikee Ryota (H. N. MEDIC), Tsunoda Masataka (H.N. MEDIC), Machida Hirofumi (Shoujyunkai Takeuchi Hospital), Shinomiya Toshihiko (Yuai Nisshin Clinic), Otsubo Yoshihiko (Cardiovascular Center, Shin-Koga Hospital), and Izumino Kiyoshi (Fujikoshi Hospital).
